# Office Men

**Published:** 1871-08

**Authors:** 


### OFFICE MEN

Multitudes of clerks and other persons in large cities come to their offices in the morning, especially in warm weather, in a state of greater or less perspiration, caused by a rapid walk to their places of business. In nine cases out of ten the first thing done is to pull off the hat, then to lay aside the coat, to be replaced by an older one, as a matter of economy; this "office coat," is almost always thinner, is certainly colder; very likely the windows and doors are open, causing more or less of a draught of air. These things combined cool the body with great rapidity, sufficient sometimes to attract unpleasant attention; but oftener the interests of business do not allow such attention, the mind is compelled away to more pressing matters; a shock has been imparted to the system, a cold has been taken, either to make an immediate impress for ill, or by cumulative influences to undermine the constitution and lay the foundation of various obscure ailments, which will burrow in the system for weeks and months and weary years, an abiding source of annoying symptoms and painful ailments, from which no permanent relief will be found except in the friendly grave.

